# Allergy immunotherapy restores airway epithelial barrier dysfunction through suppressing IL-25 -induced endoplasmic reticulum stress in asthma

**DOI:** 10.1038/s41598-018-26221-x

**Published:** 2018-05-21

**Authors:** Xiefang Yuan, Junyi Wang, Yin Li, Xiang He, Bin Niu, Dehong Wu, Nan lan, Xiaoyun Wang, Yun Zhang, Xi Dai, Xing Wang, Zhigang Liu, Guoping Li

**Affiliations:** 1 Inflammation & Allergic Diseases Research Unit, Affiliated Hospital of Southwest Medical University, Luzhou, Sichuan 646000 China; 2 Department of Respiratory Disease, the Third People’s Hospital of Chengdu, Affiliated Hospital of Southwest Jiaotong University, Chengdu, 610031 China; 3 Respiratory Disease Departments, Affiliated Hospital of Southwest Medical University, Luzhou, Sichuan 646000 China; 4 The State Key Laboratory of Respiratory Disease for Allergy at Shenzhen University, Shenzhen University School of Medicine, Shenzhen, 518060 China; 5 The First Clinic College, Chongqing Medical University, Chongqing, 401331 China

## Abstract

Constant exposure to allergen triggers destructive type 2 cell-mediated inflammation. The effect of allergen specific immunotherapy (SIT) in maintaining airway epithelial barrier function in asthma remains unknown. In the current study, we showed that SIT maintained airway epithelial homeostasis in mice exposed to *dermatophagoides farinae* (*Der f*), which induced increased expression of IL-25, endoplasmic reticulum (ER) stress and airway epithelial apoptosis. Meanwhile, SIT treatment ameliorated airway inflammatory infiltration and hyper-responsiveness in allergic mice. SIT treatment restored the airway epithelial integrity, attenuated *Der f* -induced airway epithelial ER stress and epithelial apoptosis. We also found that 4-PBA, an inhibitor of ER stress, suppressed airway epithelial ER stress and apoptosis *in vitro*. The pathological changes were partially induced by IL-25-induced ER stress, epithelial tight junction damage, and cell apoptosis in airways following allergen exposure. Furthermore, IL-25 induced ER stress in airway epithelial cells *in vitro*. The IL-25-induced airway epithelial apoptosis dependent on PERK activity was inhibited by 4-PBA. Taken together, we demonstrate that SIT is effective in allergic asthma and dependent on its depressive effect on the expression of IL-25, epithelial integrity damage, and epithelial ER stress.

## Introduction

Asthma is one of the most common chronic airway inflammatory diseases classically associated with T helper 2 (Th2) inflammations^1^. It is well-known that asthma affects approximately 300 million individuals all over the world, and the number of individuals affected worldwide will increase by 100 million people by 2025. The environmental factors may play a key role in the pathogenesis of allergic asthma^2^. Emerging evidences indicate that epithelial barrier constantly faces environmental assaults, exhibits the first line of defense against microorganisms to ensure host survival and pathogen clearance. To maintain tissue homeostasis and avoid diseases, damaged epithelial cells need to be readily replenished^3^. Airway epithelium fulfill multiple roles by maintaining homeostasis, such as maintaining airway surface liquid levels, the mucociliary escalator, epithelium restitution upon injury and barrier function in the airways^4^. The exposure to environmental allergens like house dust mite can increase oxidative stress and apoptosis in the bronchial epithelium, and can induce imbalance in airway epithelial homeostasis as a modulator of asthma pathogenesis^5^. The impaired barrier function caused by induction of imbalance in airway epithelial homeostasis allows greater access of environmental allergens, microorganisms, and toxicants to the airway tissues as well as increased production of cytokine^6^. Excess pathogens or allergens exposure to airway causes epithelial homeostasis imbalance, induces cellular stresses like endoplasmic reticulum (ER) stress, impair tight injunctions (TJs) and exacerbates epithelial apoptosis, which further severely impaired epithelial homeostasis.

It has been confirmed that allergy immunotherapy is able to revise the immunity imbalance of underlying atopic condition so as to prevent new allergic sensitization and to arrest progression of allergic rhinitis to asthma^7^. Allergen immunotherapy exhibits an inhaled corticosteroids-sparing effect for house dust mite (HDM) allergic asthmatics^8^. Standardized HDM subcutaneous immunotherapy is generally well tolerated for HDM exposure, and reduces the dosage of inhaled corticosteroid in inhaled corticosteroids treatment of HDM-allergic asthma^9^. Cockroach protease allergen disrupts airway epithelial barrier and induces allergic airway inflammation, which might be involved in airway epithelial homeostasis imbalance with high IL-33 and TSLP microenvironment^10^. Three epithelial-derived type 2 inflammation-associated cytokines, interleukin IL-25, thymicstromal lymphopoietin (TSLP) and IL-33, are important therapeutic candidates for severe asthma. IL-25 enhances steroid resistant and further exacerbates allergic disease^11^. It has been confirmed that IL-25 is critically involved in airway remodeling in response to HDM^12^. IL-25 contributes to PI3K/Akt and Erk/MAPK pathways in asthma^13^. However, the role of allergy immunotherapy in maintaining airway epithelial homeostasis and epithelial barrier function remains unclear.

In this study, we investigated that SIT contributed to maintain airway epithelial function by attenuating airway epithelial ER stress and apoptosis induced by *Der f*, and repairing the loss of ZO-1 and E-cadherin. IL-25 was primarily responsible for airway epithelial ER stress and apoptosis in an allergic asthma model. Furthermore, IL-25 induced airway epithelial apoptosis, ER stress and tight junction restoration *in vivo*, which could be inhibited by 4-PBA associated with PERK pathway.

## Methods

### Reagents

The following primary antibodies were purchased from Abcam Biotechnology: anti-BIP (Abcam, ab21685), anti-CHOP (Abcam, ab179823), anti-phospho-PERK (Abcam, ab192591), anti-PERK (Abcam, ab79483), anti-phospho-eIF2α (Abcam, ab32157), anti-ZO-1 (Abcam, ab96587), anti-E-cadherin (Abcam, ab76055), anti-IL25 (Abcam, 115672) anti-beta Actin (Abcam, ab8227), anti-Caspase-3 (Abcam, ab13847), anti-Bcl-2 (Abcam, ab59348). The mouse anti-rabbit IgG-FITC (Santa Cruz, CA, sc-2359) and mouse anti-rabbit IgG-TR (Santa Cruz, CA, sc-3917) were purchased from Santa Cruz Biotechnology. The anti-rabbit horseradish peroxidase-conjugated IgG secondary antibodies (Cell Signaling Technology, #7074) were purchased from Cell signaling Technology. Terminal deoxynucleotidyl transferase dUTP nick end labeling (TUNEL) apoptosis Kit (Abcam, ab206386) and recombinant human IL25 protein (Abcam, ab174086) was purchased from Abcam Biotechnology. The methacholine (Sigma-Aldrich, A2251), thapsigargin (Sigma-Aldrich, T9033), Fluorescein isothiocyanate–dextran (FITC-DX; Sigma-Aldrich, 46944) and 4-Phenylbutyric acid (4-PBA; Sigma-Aldrich, P21005) were purchased from Sigma-Aldrich. Mouse CD4^+^CD25^+^Treg Cells Kit (Invitrogen, 11463D) and 4′-6-diamidino-2-phenylindole dihydrochloride (DAPI, Invitrogen, D1306) were purchased from Invitrogen. IL-4 (Invitrogen, 88-7844-21), IL-5 (Invitrogen, KMC0051), IL-13 (Invitrogen, 88-7137-22), IL-17A (Invitrogen, BMS6001) and IL-25 (Invitrogen, BMS6046) ELISA Kit were purchased from Invitrogen.

### Animals and experimental protocol

The 6-8-week-old female BALB/c mice were obtained from the Animal Center of Guangdong Province and maintained under specific pathogen-free condition in the Animal Experimental Center of Shenzhen University. All animal experimental protocols and procedures in this study were approved and performed in accordance with the guidelines of the Committee of Animal Experiments Center of Shenzhen University and the National Institute of Health guidelines on the care and use of animals.

Mice were intraperitoneally sensitized on days 0, 7 and 14 with 50 μg of *dermatophagoides farinae* (*Der f*) extracts and 2 mg of aluminum hydroxide. The mice were challenged with an intranasal administration of 10 μg of *Der f* in 50 μl of phosphate buffered saline (PBS)^14^. For SIT treatment mice, the sensitized mice were given to a subcutaneous injection of 100 μg *Der f* in 100 μl of PBS 8 times at an interval of 2 days from 28–42 days. Control group of PBS, *Der f*-exposed, and 4-PBA-treated mice were given PBS. For 4-Phenylbutyric acid (4-PBA) positive control mice, 4-PBA (1 g/kg body weight per day; Sigma-Aldrich) diluted in PBS were given to sensitized mice by intragastric administration 2 hours before the challenge with *Der f* extracts.

Airway responsiveness was measured with inhaled methacholine (Sigma, The Netherlands) using Buxco whole-body plethysmography system (Buxco Research Company, United States)^15^. The enhanced pause (Penh) was used to represent airway responsiveness. Twenty-four hours after the final intranasal challenge, the mice were killed with an intraperitoneal injection of overdose pentobarbital (150 mg/kg). The mouse trachea was cannulated with a 20-gauge catheter, and the lungs were slowly lavaged with 500 μl of PBS. The total cell numbers and cell differentials were counted as described previously in bronchoalveolar lavage fluid. Supernatants were stored at −80 °C for cytokines analyses.

### Histology and TUNEL assay

Lung tissues were fixed in 10% neutral-buffered formalin for 24 h and then embedded in paraffin. The sections (5μm) of the lung specimens were stained with hematoxylin-eosin staining (H&E) methods to assess histology. For apoptosis assay, we used the TdT-mediated dUTP nick-end labeling (TUNEL) assay to detect apoptosis in lung according to previous report^16^. After treated with 0.1%Triton X-100 and Proteinase K, the sections were incubated with TUNEL reaction mixture and incubated with converter-POD. DAB was used as substrate. The stained slides were observed with a blinded fashion by two independent pulmonary observers using DM4000 Leica light microscope (Leica, Germany). The degree of peribronchial and perivascular inflammation was scored from 0 to 3 according to our previous methods, with approximately 10 areas scored in total^17^. The average percentages of positive apoptotic cells were calculated by analyzing in ten random fields from different sections.

### Measurement of cytokine levels and HDM-specific IgE in sera

Cytokine levels of IL-4, IL-5, IL-13, IL-25 in BALF and IL-10, IL-17A in serum were determined in triplicate using an enzyme-linked immunosorbent assay kits (eBioscience Co, San Diego, CA) (ELISA) according to the manufacturer’s instructions. HDM-specific IgE antibodies were measured by indirect ELISA according to our previous descriptions^17^. The results were measured by ELx808 absorbance microplate reader (BioTek, Shanghai, China) at 450 nm.

### Cell culture

The human bronchial epithelial cell (16HBE) line was obtained from a cell bank (American Type Culture Collection (*ATCC)*; Manassas, USA) and kept in our laboratory. 16HBEs were cultured with Dulbecco’s Modified Eagle’s Medium (DMEM) culture medium containing 10% fetal bovine serum (FBS) and grown to 70% confluence at 37 °C in with 5% CO2 condition. After the 16HBE cells reached 80% confluence in 6-well plastic plates, the cells were pretreated with serum-free DMEM culture medium. Several concentrations of recombinant protein IL-25 (1 ng/ml, 10 ng/ml, 100 ng/ml) were added to stimulate 16HBE cells in a concentration dependent manner.

### Flow cytometry

Mice spleen were removed, homogenized and filtered through a 200-mesh screen. Mononuclear cells (MCs) in splenocytes were isolated using lymphocyte ficoll^18^. The frequency of CD4 + CD25 + Foxp3 + Treg cells in MCs was measured to assess therapeutic effect of SIT. A number of 10^6^ cells of every sample were set to be stained with the following antibodies: fluorescein isothiocyanate (FITC)-conjugated anti-CD4, APC-conjugated anti-CD25, and phycoerythrin (PE)-conjugated anti-Foxp3 and analyzed by a FACS array (BD Bioscience, USA). Data were analyzed by software flowjo (FlowJo flow cytometry analysis software, Tree Star, USA).

### Immunohistochemical staining and Western blotting

The frozen sections of lung tissues with a thickness of 4 μm were prepared from lung embedded in optimal cutting temperature (OCT) compound. Frozen sections and cells in culture dishes were fixed with methanol and permeabilized with PBS containing 0.25% Triton X-100 for 10 min at room temperature separately. Specimens were blocked with 1% BSA in PBS containing 0.05% Tween for 1 hour, then incubated with BIP (Immunoglobulin heavy chain binding protein), CCAAT/enhancer-binding protein homologous protein (CHOP), ZO-1 antibodies at 4 °C overnight. FITC-conjugated goat anti-rabbit or TRITC-conjugated goat anti-rabbit abs were used to bind primary Abs. Nuclei were stained with 4′-6- diamidino -2-phenylindole dihydrochloride (DAPI). The primary antibody was replaced with a isotype control as the negative control. The specimens were observed by SP5 confocal microscopy (Leica, Germany) and analyzed with Leica Application Suite Software.

For western blotting, lung tissues and cells were homogenized and solubilized in cold radioimmunoprecipitation assay (RIPA) buffer (20 mM Tris-HCl, pH 7.5, 150 mM NaCl, 1 mM EDTA, 1 mM EGTA, 1% NP-40, 1% sodium deoxycholate) with an optional protease inhibitor cocktail. Protein concentrations were detected by Epoch-volume spectrophotometer system (Biotech, USA). Proteins were separated by electrophoresis in 10% SDS-PAGE and transfered to a microporous polyvinylidene difluoride (PVDF) membrane at 100 mA for 2 hours using a wet transfer method. After incubated in 5% nonfat milk in Tris-buffered saline/Tween for 1 h, the membranes were incubated with PERK, p-PERK, p-eIF2α, BIP, CHOP, β-actin, Caspase-3, Bcl-2 antibody (1:1000 dilution in TBST) at 4 °C overnight. Anti-rabbit horseradish peroxidase-conjugated IgG secondary antibodies were used to bind primary antibodies. And bands were detected by using ECL Western Blotting kit.

### RNA isolation and real-time PCR

RNA was isolated from lung tissue using the TRIzol RNA Reagent according to the manufacturer’s instructions (Invitrogen, Thermo Scientific, USA), which was reverse-transcribed to cDNA using PrimeScript^TM^ RT Master Mix (Takara, China). qPCR was performed using SYBR Advantage qPCR Premix (Clontech, USA). The 2–2-ΔΔCT threshold method was used to calculate relative quantitative levels of individual gene. The primer sequences were as follows: GAPDH primer (sense, 5′-AAGAAGGTGGTGAAGCAGG-3′; antisense, 5′-GAAGGTGGAAGA GTGGG AGT-3′; CHOP (sense, 5′ TCAGCCCACCGTAACAAT 3′; antisense 5′ CAAACTT CTCGGCGTCAT 3′).

### Assessing cells apoptosis

16HBE cells were seeded in 6 well plates and treated with treated with IL-25 (100 ng/ml), thapsigargin (Tg, 0.5uM) and 4-PBA (5 mM; Sigma, St Louis, USA) respectively in a serum-free culture medium. After treatment 2 hours with 4-PBA, the medium containing IL-25 (100 ng/ml) was added to co-incubate cells. Following treatment, the cells were harvested by 0.2% trypsin. 10^6^ cells were suspended in 100ul binding buffer with 5ul Annexin V-FITC and 5ul PI staining solution for 10 min at room temperature. The samples were set to analysed by a FACS array. Data were analyzed by software flowjo (FlowJo flow cytometry analysis software, Tree Star, USA).

### Airway epithelial barrier measurement

16HBE cells were seeded at a density of 2 × 10^5^ cells in 1.13-cm2, 0.4-μm permeable polyester inserts (Corning) and complete confluent monolayers of cells were reached for 3 additional days. IL-25 (100 ng/ml), Tg (0.5 μM), 4-pba (5 mM, 2 h before IL-25 stimulation) were added to the apical chamber. After 24 h, trans endothelial electrical resistance (TEER) values were detected by using a commercial ohmmeter (EVOM, WPI, USA).

Paracellular flux of 4-kDa FITC-DX across confluent monolayers of 16HBE cells was detected as previously^19^. 16HBE cells were seeded at a density of 1 × 10^5^ cells in 24-well transwell inserts and confluent monolayers of cells were reached for 3 additional days. 3 mM FITC-DX was added to apical chamber. After incubation for 3 h at 37 °C, samples from apical and basal chambers were harvested for FITC-DX concertration detection every 30 min for 90 min. The rate of FITC-DX flux was calculated by the following formula: P_o_ = [(F_A_/∆t)V_A_]/(F_L_A). where P_o_ is in centimeters per second; F_A_ is basal chamber fluorescence; F_L_ is apical chamber fluorescence; ∆t is change in time; A is the surface area of the filter (in square centimeters); and V_A_ is the volume of the basal chamber (in cubic centimeters).

### Statistical analysis

All statistical data were analyzed by the SPSS 17.0 software (IL, USA). Results are presented as means ± s.d. Significant differences between two groups were determined with the Tukey-Kramer post-test or Dunnett’s T3 method. Differences among multiple groups were measured by using one-way ANOVA. *P* values < 0.05 was considered as a significant criterion.

## Results

### SIT and 4-PBA attenuated airway hyper-responsiveness and airway inflammation

**Figure 1:**
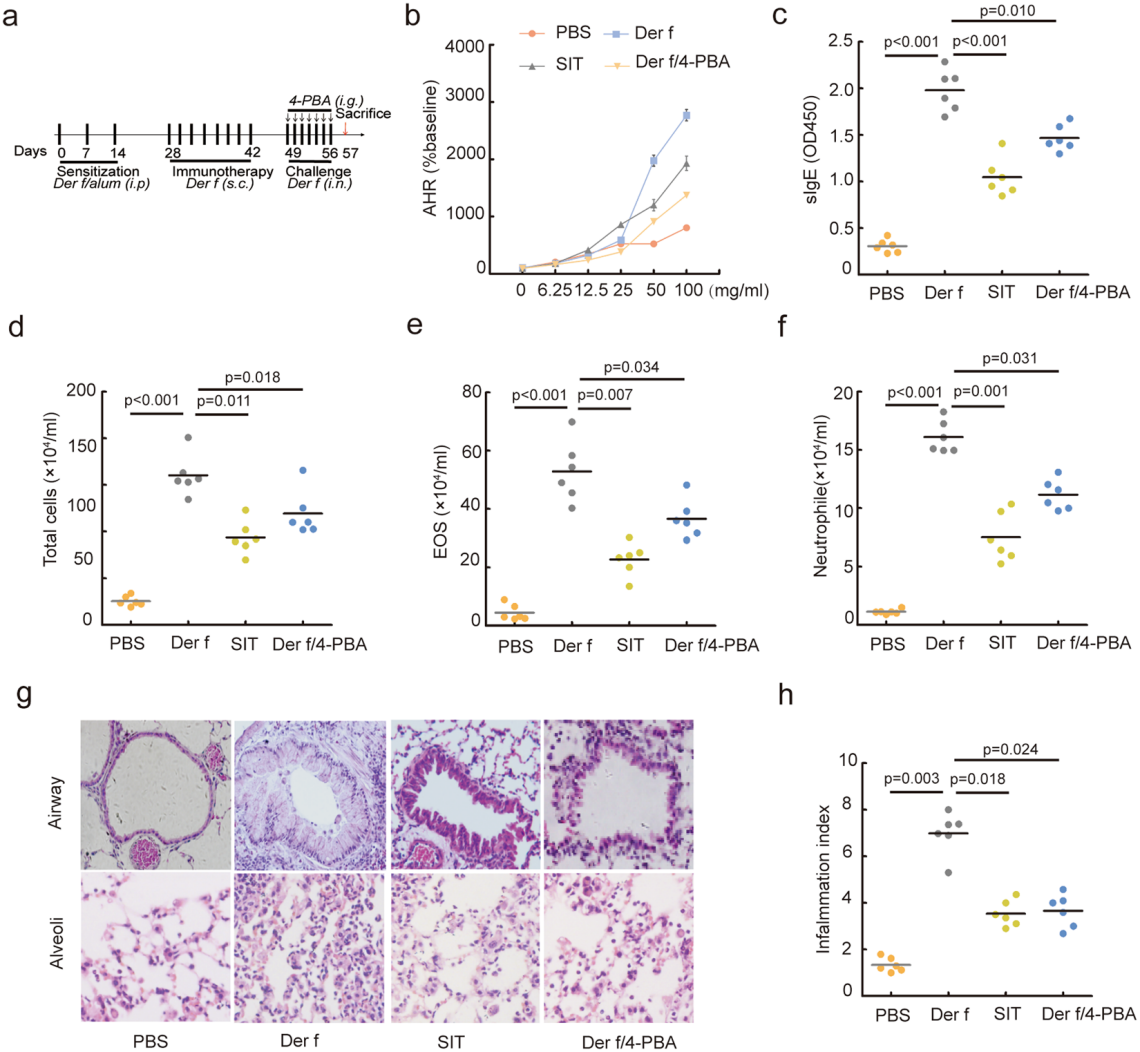
Specific-allergen immunotherapy attenuates airway inflammation and airway hyperresponsiveness in Der f-sensitized mice. (**a**) Experimental design for Der f-specific immunotherapy and 4-PBA treatment in Der f extracts-induced allergic mice (n = 8). (**b**) AHR was assessed by measured the enhanced pause (Penh) (n = 6). (**c**) Serum Der f-specific IgE was measured using optical density (OD) by ELISA. Total inflammatory cells (**d**), eosinophils (**e**), neutrophils (**f**) separated from BALF were counted by differential cell analysis using Wright’s staining. (**g**) The airway and alveoli tissue sections were stained by H&E (original magnification, ×200). (**h**) The degree of inflammation in airway was scored by Kruskal-Wallis test. All data were represented as means ± SEM (n = 6, one-way ANOVA, significant differences were defined as p < 0.05).

Previous studies have proved that SIT could alleviate inflammatory response and clinic symptoms in asthmatic patients^20^. ER stress plays a critical role in pathogenesis of asthma^21^. However, it remains unclear whether SIT inhibits airway inflammation and airway hyper-responsiveness by regulating ER stress. To investigate the effect of SIT in ER stress, the ER stress inhibitor 4-PBA was used as a positive control to evaluate the role of SIT in allergic mouse model. *Der f* -sensitized mice received a subcutaneous immunotherapy with *Der f* (Fig. 1a). In our present studies, we observed that *Der f* exposure led to an increasing of bronchial responsiveness to methacholine in *Der f*-induced mice, while SIT significantly suppressed bronchial responsiveness to methacholine in *Der f*-induced mice. At a methacholine dose of 0, 6.25, 12.5, 25, 50 and 100 mg/ml, Penh values in *Der f*-induced mice treated with SIT (SIT mice) were significantly decreased compared to *Der f*-induced mice. In presence of 4-PBA, we observed that 4-PBA treatment significantly decreased Penh values in *Der f*-induced mice treated with 4-PBA at a methacholine dose of 50 and 100 mg/mL compared to *Der f*-induced mice (Fig. 1b, *p* < *0*.*001*). SIT inhibited the levels of *Der f* -specific IgE significantly in SIT mice compared to *Der f*-induced mice (Fig. 1c, *p* < *0*.*001*). And 4-PBA treatment did significantly inhibit the levels of *Der f*-specific IgE (sIgE) in *Der f*-induced mice (Fig. 1c, *p* = *0*.*010*). We found that SIT and 4-PBA resulted in a significant decrease in total inflammatory cells (Fig. 1d, *p* = *0*.*011* and *p* = *0*.*018*), eosinophils (Fig. 1e, *p* = *0*.*007* and *p* = *0*.*34*) and neutrophils (Fig. 1f, *p* = *0*.*001* and *p* = *0*.*031*) in the BAL fluid compared to *Der f*-induced mice. Histological analysis revealed that inflammatory cell infiltration was decreased in the airway, around blood vessels and in the alveoli of SIT mice and 4PBA-treated mice (Fig. 1g). There was a reduction of inflammation index in the lung of SIT mice compared to *Der f*-induced mice (Fig. 1h, *p* = *0*.*018*). The inflammation index in 4-PBA-treated mice was significantly decreased by 42.03% (2.83/6.73) compared to *Der f*-induced mice (Fig. 1h, *p* = *0*.*024*). Taken together, these results suggested that SIT and 4-PBA attenuated airway hyper-responsiveness and airway inflammation.

### SIT suppressed Der f -induced ER stress and PERK activity in mice

**Figure 2:**
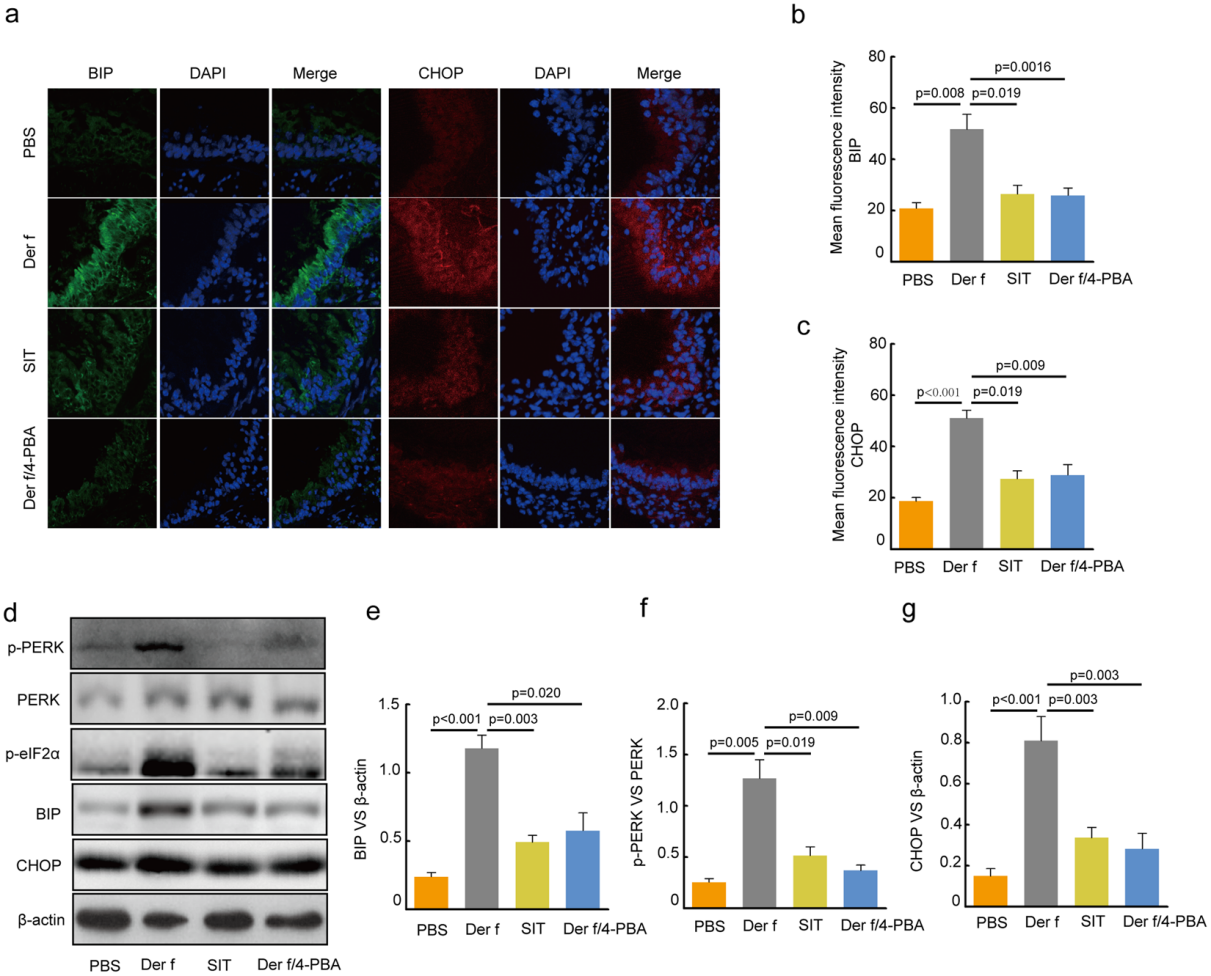
Specific-allergen Immunotherapy reduces ERs in Der f-sensitized mice. (**a**) The expression of BIP and CHOP was measured using immune staining. DAPI was used for staining nucleus. (**b**,**c**) Fluorescence intensity of BIP and CHOP at airway location was measured 10 times at random fields in each group. (**d**) Expression of p-PERK, PERK, p-eIF2α, BIP, CHOP in lung tissue were determined by western blot. (**e**–**g**) Relative changes in the density of BIP, CHOP and β-actin, p-PERK and PERK were analysed. Blots in panel (**d**) have been cropped, and full blots are presented in Supplementary Fig. S2. All data were represented as means ± SEM (n = 6, one-way ANOVA, significant differences were defined as p < 0.05).

The role of ER stress in asthma has been confirmed^1,21,22^. However, the effect of SIT in ER stress remains unelucidated. To investigate whether SIT suppresses *Der f*-induced ER stress in mice exposed to *Der f*, ER stress markers in lung tissue were determined by immunofluorescence staining. 4-PBA was also used as a positive control for ER stress. Consistent with our previous studies, the expression and localization of BIP and CHOP predominantly occurred in the cytoplasmic areas of the airway epithelium in *Der f*-induced mice. We found that SIT significantly inhibited the levels of BIP and CHOP in SIT mice compared to *Der f*-induced mice (Fig. 2a). The immunofluorescence intensities of BIP and CHOP in lung of SIT mice were reduced by 49.93% (25.86 /51.79) and 43.38% (22.16/51.08) respectively compared to those in *Der f*-induced mice (Fig. 2b,c, *p* = *0*.*019* and *p* = *0*.*005*). In addition, western blot also exhibited similar reductions of BIP and CHOP in SIT mice compared to those in *Der f*-induced mice (Fig. 2d–f, *p* = *0*.*003* and *p* = *0*.*003*). Furthermore, we found that 4-PBA significantly repressed the levels of BIP and CHOP in *Der f*-induced mice, consistent with SIT (Fig. 2d–f, *p* = *0*.*020* and *p* = *0*.*003*).

PERK (protein kinase-like kinase) is a key ER stress sensor of unfolded protein response^23^. Sustained PERK/CHOP signaling induces apoptosis process under prolonged ER stress circumstance^24,25^. We showed that the levels of p-eIF2α and p-PERK was increased in *Der f*-induced mice after *Der f* exposure. The expression of PERK in *Der f*-induced mice was not suppressed by neither SIT nor 4-PBA. However, the phosphorylation of PERK in SIT mice and 4-PBA treatment mice was significantly decreased (Fig. 2d, g *p* = *0*.*019* and *p* = *0*.*009*). These results supported the hypothesis that SIT treatment repressed ER stress and PERK activity.

### SIT inhibited the levels of IL-25 with inducing CD4^+^CD25^+^ Foxp3^+^ Treg cells

**Figure 3:**
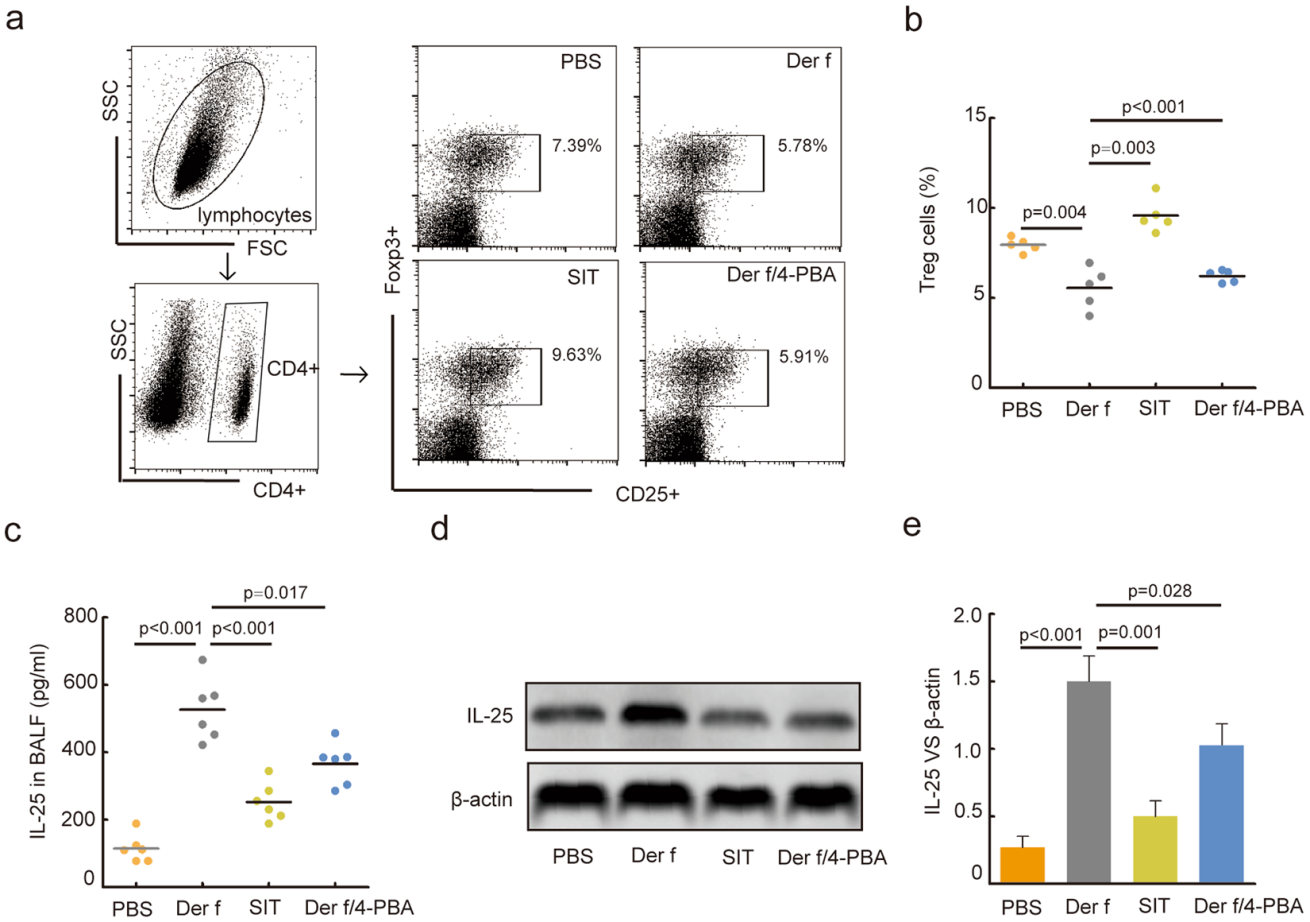
Specific-allergen Immunotherapy increases the level of Treg cells and decreases level of IL-25. (**a**) CD4+ CD25+ Foxp3+ Treg cells in spleen tissue of mice were detected using flow cytometry. (**b**) The percentage of CD4+ CD25+ Foxp3+ Treg cells of each mice were shown in the scatter plot. 8 × 10^4^ splenocytes were analysed in each sample. (**c**) The expression of IL-25 in BALF was measured using ELISA. (**d**) The expression of IL-25 in lung tissue was detected by western blots. (**e**) Relative changes in the density of IL-25 and β-actin was analysed. Blots in panel (d) have been cropped, and full blots are presented in Supplementary Fig. S3. All data were represented as means ± SEM (n = 6, one-way ANOVA with Tukey’s post hoc, significant differences were defined as p < 0.05).

Previous studies have demonstrated that Treg cell ameliorated airway inflammation through IL-10^26^. As previously shown^27^, ER stress drives a regulatory phenotype in human T-cell clones. To investigate the effects of Treg cells and IL-25 in airway inflammation in ER stress, 4-PBA was also used as an ER stress inhibitor *in vivo*. As demonstrated by flow cytometry, *Der f* exposure inhibited the frequencies of CD4^+^CD25^+^Foxp3^+^ Treg cell with an increasing of IL-25. The frequencies of CD4^+^CD25^+^ Foxp3^+^ Treg cell in spleen tissue were markedly enhanced in SIT mice compared to *Der f*-induced mice. SIT and 4-PBA significantly decreased the level of IL-25 in BALF in *Der f*-induced mice (Fig. 3c–e). We also noted that SIT and 4-PBA suppressed the levels of Th2 cytokines (IL-4, IL-5 and IL-13) in BALF from *Der f*-induced mice (Fig. S1). The results indicated that SIT inhibited IL-25 and Th2 cytokines with inducing CD4^+^CD25^+^ Foxp3^+^ Treg cells. ER-stress may be involved in the changes of immune phenotype in *Der f*-induced mice.

### SIT restored airway epithelial barrier dysfunction

**Figure 4:**
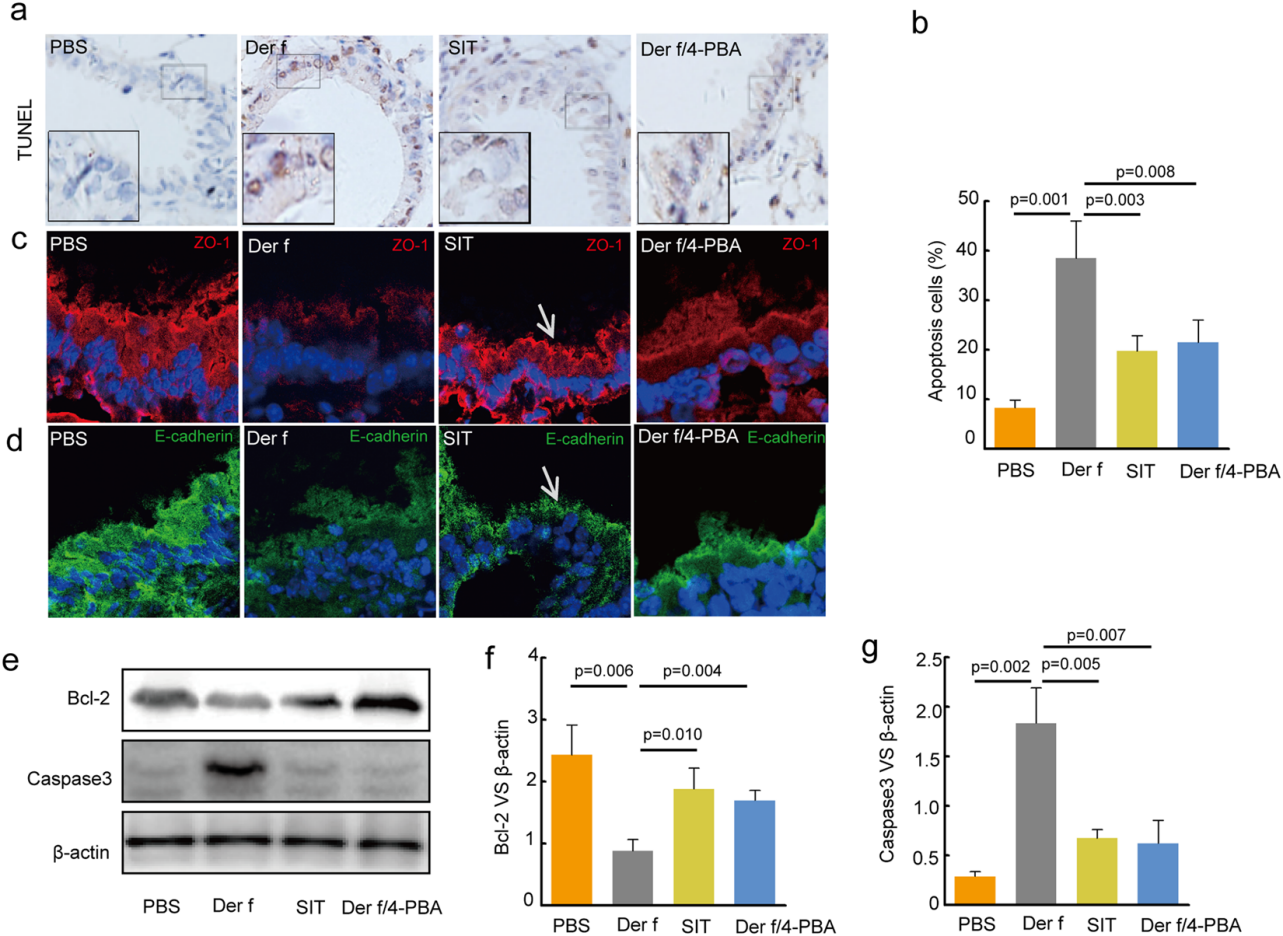
Specific-allergen Immunotherapy decrease airway epithelial apoptosis and repairs impaired epithelial tight junction in Der f-sensitized mice. (**a**) Epithelial apoptosis in airway of mice was detected by TUNEL. Nucleus stained by substrate diaminobenzidine (DAB) represent apoptosis occurrence. (original magnification, ×200). (**b**) The percentage of apoptosis cells in airway was measured at 10 times at random fields in each group. (**c**,**d**) Fluorescence intensity of tight junction protein ZO-1 (arrow), E-cadherin was observed by confocal laser scanning microscopy. (**e**) Expression of Bcl-2, Caspase-3, β-actin in lung tissue were measured by western blot. (**f**,**g**) Relative changes in the density of Bcl-2, Caspase-3 and β-actin were analysed. Blots in panel (**e**) have been cropped, and full blots are presented in Supplementary Fig. S4. All data were represented as means ± SEM (n = 6, one-way ANOVA with Tukey’s post hoc, significant differences were defined as p < 0.05).

Airway epithelial barrier plays a key role in preventing access of the inspired luminal contents to the sub-epithelium^28^. The apoptosis of airway epithelial cells could aggravated allergic features^29^. Therefore, to determine whether SIT affects airway epithelial barrier function in asthma, we observed the level of tight junction protein zonula occludens-1(ZO-1), E-cadherin and apoptosis of airway epithelial cells. We found an increase number of TUNEL-positive airway epithelial cells in *Der f*-induced mice, while the number of TUNEL-positive airway epithelial cells was decreased by approximately 48.7% in SIT mice (Fig. 4a,b). Moreover, the level of caspase-3 in SIT mice was decreased, but the level of bcl-2 was increased compare with that in *Der f*-induced mice (Fig. 4e–g, *p* = *0*.*005* and *p* = *0*.*010*). Furthermore, TUNEL-positive airway epithelial cells were decreased by approximately 44.2% in the presence of ER stress inhibitor 4-PBA, with an increasing level of bcl-2 and a decreased level of caspase-3. Tight junction protein ZO-1 and E-cadherin are components of airway epithelial barrier. We observed the exaggerated loss of ZO-1 and E-cadherin in *Der f*-induced mice. However, SIT treatment promoted the production of ZO-1 and E-cadherin. We also observed that the level of ZO-1 and E-cadherin was further enhanced in the presence of 4-PBA (Fig. 4c,d). Thus, SIT and 4-PBA restored airway epithelial barrier dysfunction, which was induced by exposure to HDM.

### IL-25 induced ER-stress response in 16HBE cells

**Figure 5:**
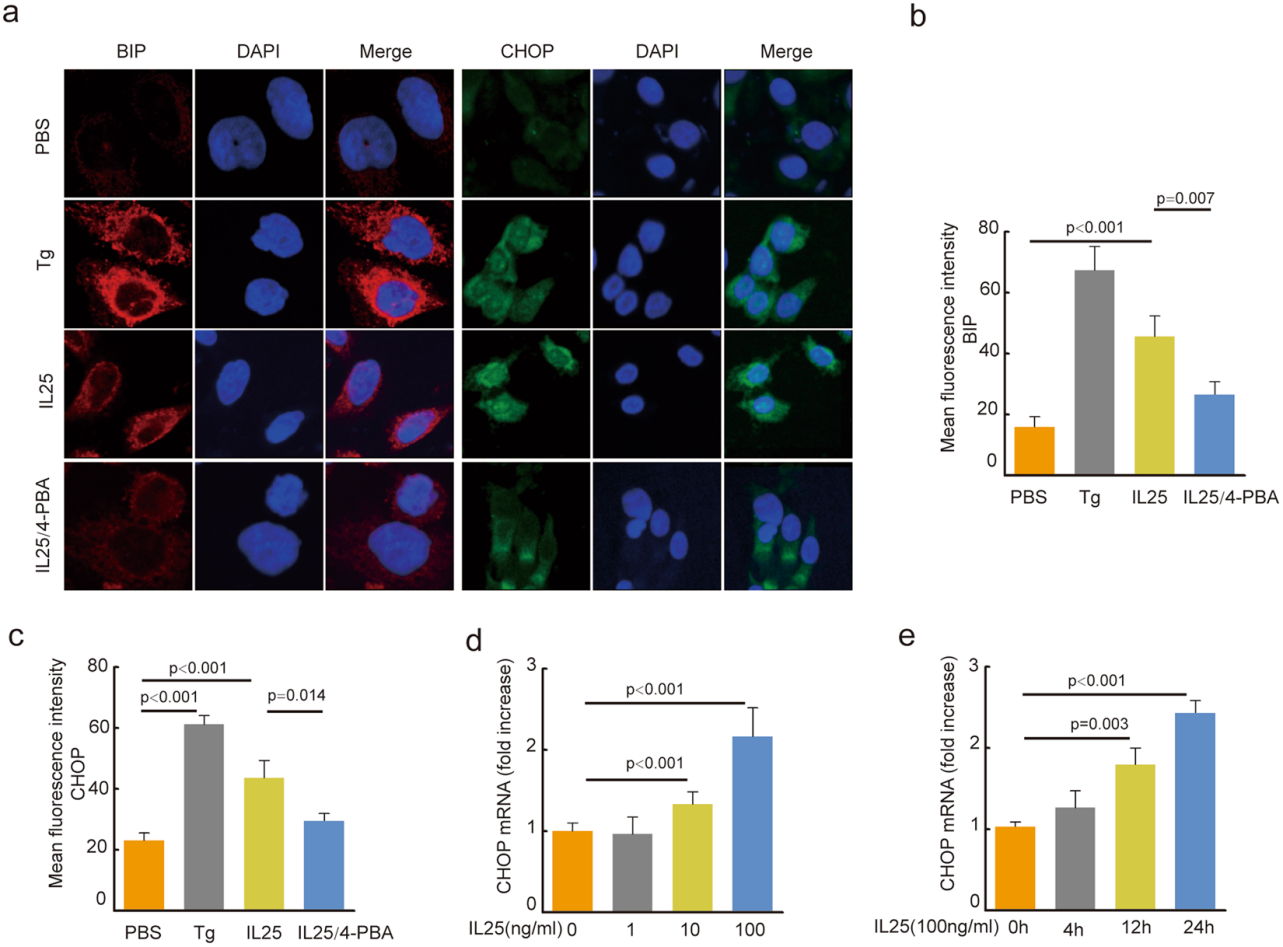
IL-25 induces ERs in 16HBE cells. (**a**) Expression of BIP and CHOP were measured by immunofluorescent staining with BIP and CHOP antibody. DAPI was used for staining nucleus. TRICT-conjugated secondary abs (red) and FITC-conjugated secondary abs (green) were used for binding confocal laser scanning microscopy (original magnification, ×400). Fluorescence intensity of BIP (**b**) and CHOP (**c**) were quantified at 10 random fields. The mRNA levels of CHOP induced by IL25 were detected at a dose-dependent manner (**d**) (0 ng/ml, 1 ng/ml, 10 ng/ml, 100 ng/ml) and a time-dependent manner (**e**) (0 h, 4 h, 12 h, 24 h). All data were represented as means ± SEM (n = 6, one-way ANOVA with Tukey’s post hoc, significant differences were defined as p < 0.05).

In present studies, we found that SIT could inhibit IL-25 and alleviate ER stress in lung of *Der f*-induced mice. To further investigate whether IL-25 directly induces ER stress in airway epithelial cells, we also observed the effect of IL-25 on expression and localization of BIP and CHOP in 16HBE cells. Thapsigargin (Tg), an ER stress inducer, was used as positive control. Confocal microscopic analyses revealed that the expression of ER stress response marker BIP was significantly decreased, and predominantly localized in cytoplasmic areas in IL-25-treated 16HBE cells compared to PBS control cells (Fig. 5a,b). The expression of BIP was also inhibited by 4-PBA in IL-25-treated 16HBE cells (Fig. 5a,b *p* = *0*.*007*). Previous studies showed that CHOP is an ER stress response factor or a proapoptotic player in response to ER stress^30^. We found that the expression of CHOP was increased by IL-25 treatment, and the translocation of CHOP from cytoplasm to nucleus was induced by IL-25 treatment as well (Fig. 5a,b, *p* < *0*.*001*). Moreover, IL-25-induced CHOP expression and translocation to nucleus were inhibited by 4-PBA (Fig. 5a,c, *p* = *0*.*014*). We further observed the effect of IL-25 on CHOP transcription level. In the presence of 1, 10 and 100 ng/ml IL-25, we found that the elevated mRNA expression levels of CHOP in 16HBE cells exhibited a dose-dependent manner in 16HBE cells (Fig. 5d). The elevated mRNA expression levels of CHOP were a time-dependent manner in 16HBE cells treated with 100 ng/ml IL-25 (Fig. 5e). Together, these data indicated that IL-25 induced ER-stress response and triggered the transcription factor CHOP.

### IL-25 caused airway epithelial barrier dysfunction with epithelial apoptosis

**Figure 6:**
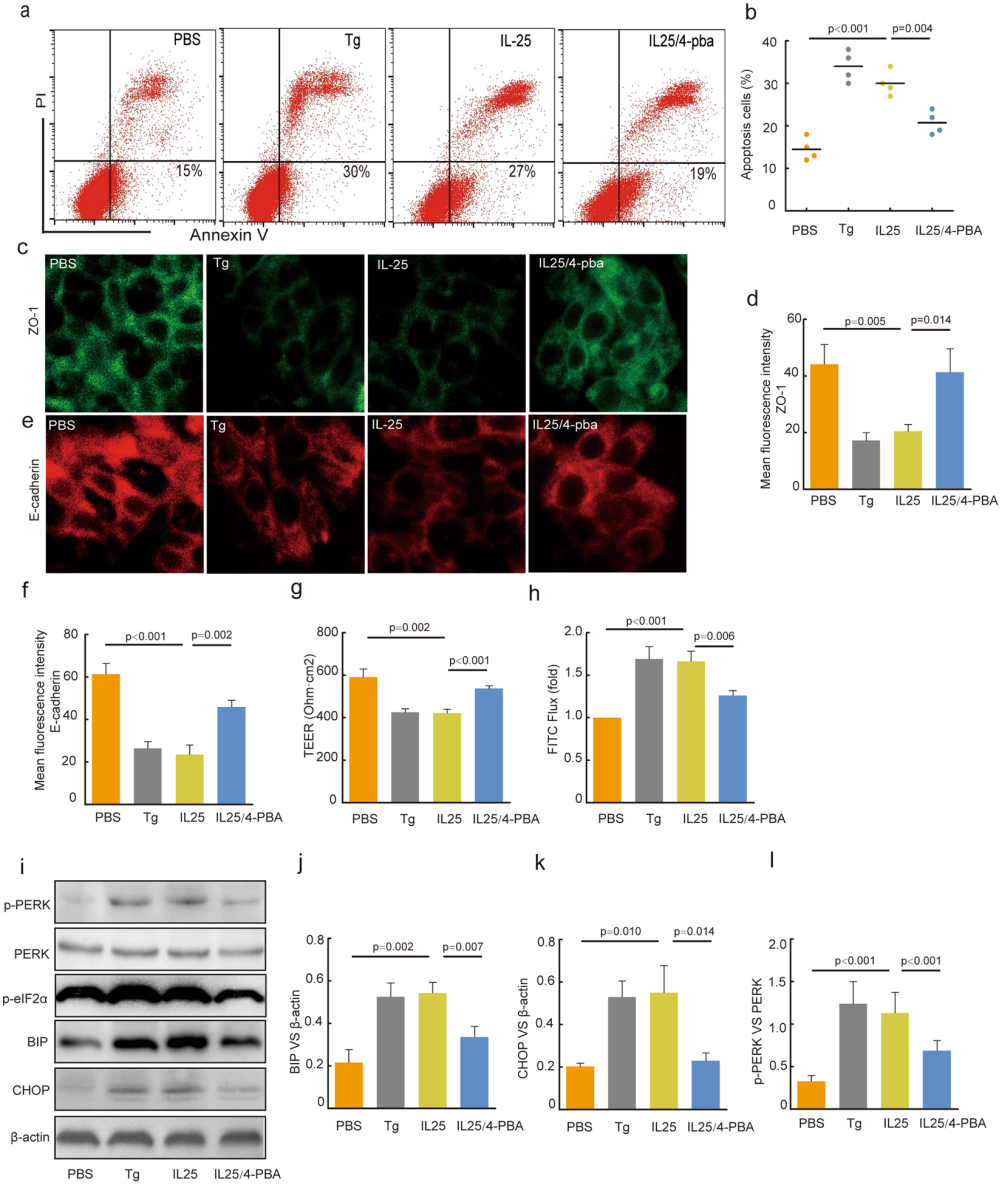
4-PBA inhibits IL-25-induced epithelial barrier damage and suppresses apoptosis of 16HBE cells associated with PERK pathway. (**a**) The apoptotic cells were determined by flow cytometry using Annexin V/Propidium iodide (PI) staining. (**b**) The percentages of apoptotic cells were analysed. (**c**–**f**) Confocal laser immunofluorescence photomicrographs of ZO-1 and E-cadherin were measured (original magnification, ×400). Epithelial barrier function was detected by TEER measurement (**g**) and FITC-DX paracellular flux assay (**h**,**i**) The expression of p-PERK, PERK, p-eIF2α, BIP and CHOP were measured using immunoblotting. Relative changes in the density of BIP (**j**), CHOP (**k**) and β-actin, p-PERK and PERK (**l**) were analysed. Blots in panel (**g**) have been cropped, and full blots are presented in Supplementary Fig. S5. Data were represented as means ± SEM (n = 6, one-way ANOVA with Tukey’s post hoc, significant differences were defined as p < 0.05).

The epithelial barrier dysfunction increases the susceptibility of airways in asthmatic subjects to environmental agents^31^. To further identify whether IL-25 impairs epithelial barrier function, we analyzed ZO-1, E-cadherin, TEER, rate of FITC-flux and the apoptosis of epithelial cells. Flow cytometry analysis indicated that the ER stress-induced apoptosis was increased by ER stress activator TG. IL-25 resulted in an increased apoptosis level of epithelial cells, which was suppressed by ER stress inhibitor 4-PBA compared to IL-25–treated cells (Fig. 6a,b, *p* = *0*.*004*). There was a significant decrease in TEER of Tg-treated and IL-25-treated 16HBE cells compare to control cells (Fig. 6g, *p* = *0*.*002* and *p* < *0*.*001*). Then we also found an increase in rate of paracellular flux in Tg-treated and IL-25-treated cells when compared with control cells. And the effect of Tg and IL-25 on TEER and FITC flux in 16HBE cells could be reversed by 4-PBA (Fig. 6g,h).

Western blot analysis showed that Tg increased the abundance of CHOP, BIP, p-eIF2α and p-PERK. Similar observations were made in 16HBE cells treated with IL-25. 4-PBA decreased the abundance of CHOP, BIP, p-eIF2α, and p-PERK (Fig. 6i–l). These data are consistent with our results that IL-25 acts as a regulator of epithelial barrier function. We observed that a significant decrease in ZO-1 and E-cadherin in IL-25–treated 16HBE cells. 4-PBA induced a significant increase in ZO-1 and E-cadherin in IL-25–treated 16HBE cells (Fig. 6c,e).

Together, these results show that IL-25 is a key effector of airway epithelial barrier dysfunction through ER stress-induced apoptosis of epithelial cells and reduced expression of ZO-1 associated with PERK pathway.

## Discussion

Allergen immunotherapy has been shown to effectively prevent asthma in patients with allergic rhinitis and the onset of new sensitizations^32^. T-regulatory cells and blocking antibodies play important roles in allergy immunotherapy^7^. However, 5–10% asthmatic patients exhibited poor responds to the currently available treatments such as inhaled corticosteroids and beta-adrenergic agonists. ER stress is implicated in asthma pathogenesis, and the major ER stress signaling pathway involves ER stress sensor PERK and CHOP signaling^33^. The phosphorylation/expression of PERK is one of important makers in canonical unfolded protein response (UPR) -activation. Human neutrophil elastase led to ER stress-induced apoptosis of endothelial cell by activating the PERK-CHOP branch of the unfolded protein response^34^. In this report, we described a novel role for the allergy immunotherapy in HDM-induced airway inflammation that was associated with ER stress. We demonstrated that HDM exposure induced allergic airway inflammation and airway hyper-responsiveness by significantly enhancing BIP and PERK/CHOP signaling. In addition, allergy immunotherapy led to a significant decrease in ER stress and PERK/CHOP signaling, increasing CD4^+^CD25^+^Foxp3^+^ Treg cells, and ameliorated airway hyper-responsiveness and airway inflammation.

IL-25, a distinct member of IL-17 cytokine family, regulates adaptive immunity and augments the allergic inflammation by enhancing the maintenance and functions of adaptive Th2 memory cells^35,36^. IL-17A belongs to Th17 cytokine family, and plays a key role in severe asthma. However, in this study, the expression of IL-17A in SIT mice and *Der f* had no significant change compared to PBS mice (Supplemental Fig. 1e). As one of Th2 cytokine promoter, IL-25 is produced by a series of cell types such as airway epithelial cell, eosnophils and mast cells^37^. IL-25 can exacerbate airway hyper-responsiveness and inflammatory infiltration in asthmatic patients with acute exacerbation^38,39^. Previous studies shown that allergy immunotherapy regulated the immune responses from Th2 to Th1/Treg pattern^40,41^. Most importantly, Treg cells is a critical underlying mechanism of allergy immunotherapy in allergic asthma^42^. We found that *Der f* exposure increased IL-25 and TH2 cytokines IL-4, IL-5 and IL-13 with low CD4^+^CD25^+^ Foxp3^+^ Treg cells. Furthermore, allergy immunotherapy inhibited the increasing of IL-25 and Th2 cytokines IL-4, IL-5 and IL-13 with induction of CD4^+^CD25^+^ Foxp3^+^ Treg cells. It is interesting to note that ER-stress may be involved in the changes of immune phenotype in mice induced by *Der f*. The low production of IL-25 may be one of the important factors contributing to the suppression of allergic airway inflammation and airway hyper-responsiveness in allergy immunotherapy.

Epithelial barrier plays a key role in maintaining immune homeostasis at epithelial location. In allergic asthma, the homeostasis balance of the epithelial barrier is characterized by loss of differentiation, reduced junctional integrity, and overwhelming stresses. Disruption of epithelial tight junction has been reported in asthma with low expression of claudin-18 and E-cadherin^43,44^. Moreover, house dust mite exposures impair integrity of the barrier through inhibiting activity of protease, and triggers epithelial immune response^45^. ZO-1 is involved in leak pathway, one of two transport pathways in tight junction associated with impaired epithelial function in allergic rhinitis^46^. E-cadherin also plays key role in tight junction. Indeed, we showed that repeated HDM exposure led to loss of ZO-1, E-cadherin, and induced the apoptosis of airway epithelial cells in allergic mice model. Importantly, we found that allergy immunotherapy increased in expression of ZO-1and E-cadherin without a low apoptosis of airway epithelial cells, resulting in increasing expression of anti-apoptotic Bcl-2 and inhibition of caspase-3.

Excessive ER stress affects oxygen species generation, activates inflammatory response and induces cell apoptosis through CHOP^47^. Inflammatory mediators and activation of cellular stress pathways may impact ER function, which may depend on the cell type^48^. In this study, we found that IL-25 was a potent inducer of ER stress in epithelial cells by increasing BIP and CHOP. We also found that in the presence of 4-PBA, IL-25-induced CHOP expression and translocation of CHOP were decreased. PERK-CHOP signaling regulated ER stress-induced apoptosis^49^. CHOP is regarded as a key inducer of apoptosis^33^. In our studies, IL-25 increased apoptosis level of epithelial cells, which was consistent with ER stress activator Tg-induced apoptosis. Furthermore, IL-25-induced apoptosis of epithelial cells was inhibited by 4-PBA, an ER stress inhibitor. Mechanistically, we showed that 4-PBA blocked phosphorylation of PERK and e-IF2α, suppressed expression of BIP and CHOP. The results indicated that 4-PBA could inhibit UPR -activation. Specifically, we also saw a reduced expression of tight junction marker ZO-1 and E-cadherin, which was consistent with IL-25-induced apoptosis of epithelial cells. The expression of ZO-1 and E-cadherin were significantly increased by 4-PBA in 16HBE cells treated by IL-25. The decreased expression of ZO-1, E-cadherin and increased apoptosis rate in 16HBE cells induced by IL25 may impair epithelial barrier function, which was proved by TEER measurement and FITC flux assay.

In this study, the effectiveness of immunotherapy had been related to less epithelial barrier dysfunction and ER stress through reducing the expression of an epithelial-drived cytokine, IL-25. However, the mechanism that has been associated with reducing expression level of IL-25 in SIT-treated mice need to be studied further.

Taken together, our results demonstrated that SIT alleviate ER stress, apoptosis aggravation and tight junction damage by inhibition of IL-25 expression. Our data also supported the involvement of IL-25 in apoptosis induced by ER stress, reduction of ZO-1 and E-cadherin in human bronchial epithelial cells, which is associated with the PERK/CHOP pathway. Based on these findings, we elucidated not only a novel mechanism that SIT treatment alleviates apoptosis induced by ER stress in airway epithelial by suppressing the production of IL-25, but also discovered a potential therapeutic candidate for allergic asthma.

## Electronic supplementary material

Supplementary information
