# Supplementary material for: Allergy immunotherapy restores airway epithelial barrier dysfunction through suppressing IL-25 -induced endoplasmic reticulum stress in asthma
Source: Sci Rep. 2018 May 21;8:7950. doi: 10.1038/s41598-018-26221-x (PMC5962552; doi:10.1038/s41598-018-26221-x)
Supplement: Supplementary file 1 — Supplementary information [file 41598_2018_26221_MOESM1_ESM.doc]

**Supplementary information**

Allergy immunotherapy restores airway epithelial barrier dysfunction through suppressing IL-25 -induced endoplasmic reticulum stress in asthma

Xiefang Yuan^1^,Junyi Wang^2^,Yin Li^5^, Xiang He^1^, Bin Niu^3^, Dehong Wu^2^, Nan lan^3^,Xiaoyun Wang^1^, Yun Zhang^1^,Xi Dai^1^,Xing Wang^1^,Zhigang Liu^4*^, Guoping Li^1,2*^

^1^ Inflammation & Allergic Diseases Research Unit, Affiliated Hospital of Southwest Medical University, Luzhou, Sichuan 646000, China

^2^ Department of Respiratory Disease, the Third People's Hospital of Chengdu, Affiliated Hospital of Southwest Jiaotong University, Chengdu 610031, China

^3^ Respiratory Disease Departments, Affiliated Hospital of Southwest Medical University, Luzhou, Sichuan 646000, China

^4^The State Key Laboratory of Respiratory Disease for Allergy at Shenzhen University, Shenzhen University School of Medicine, Shenzhen 518060, China.

^5^The First Clinic College, Chongqing Medical University, Chongqing 401331, China

^*^corresponding authors: Guoping Li, E-mail: [lzlgp@163.com](mailto:lzlgp@163.com), Tel: +868303165324.Fax: +868303165324; Zhigang Liu, E-mail: lzg@szu.edu.cn, Tel: 8675526681907. Fax: 8675526681906.

Running Title: **Allergy immunotherapy restores airway epithelial barrier dysfunction**


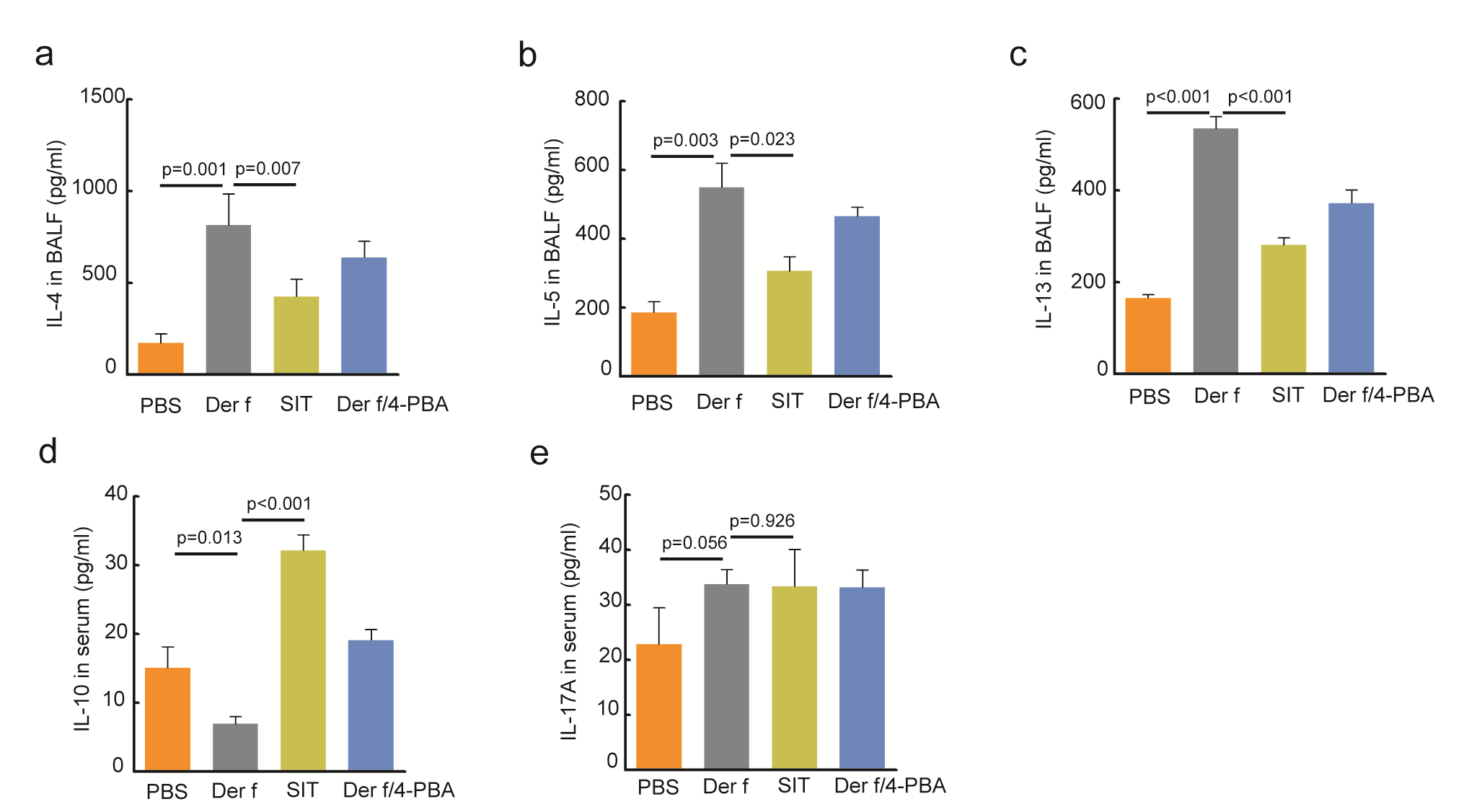


Fig S1: (a-c) Specific-allergen Immunotherapy decreases Th2 cytokines in Der f-sensitized mice. The expression of IL-4, IL-5, IL-13 in BALF were measured using ELISA. (d) Specific-allergen Immunotherapy promotes production of IL-10 in serum. The expression of IL-10 was measured using ELISA. (e) The expression of IL-17A in serum was assessed using ELISA. Data were represented as means ± SEM (n=3, one-way ANOVA with Tukey’s post hoc, significant differences were defined as p＜0.05)


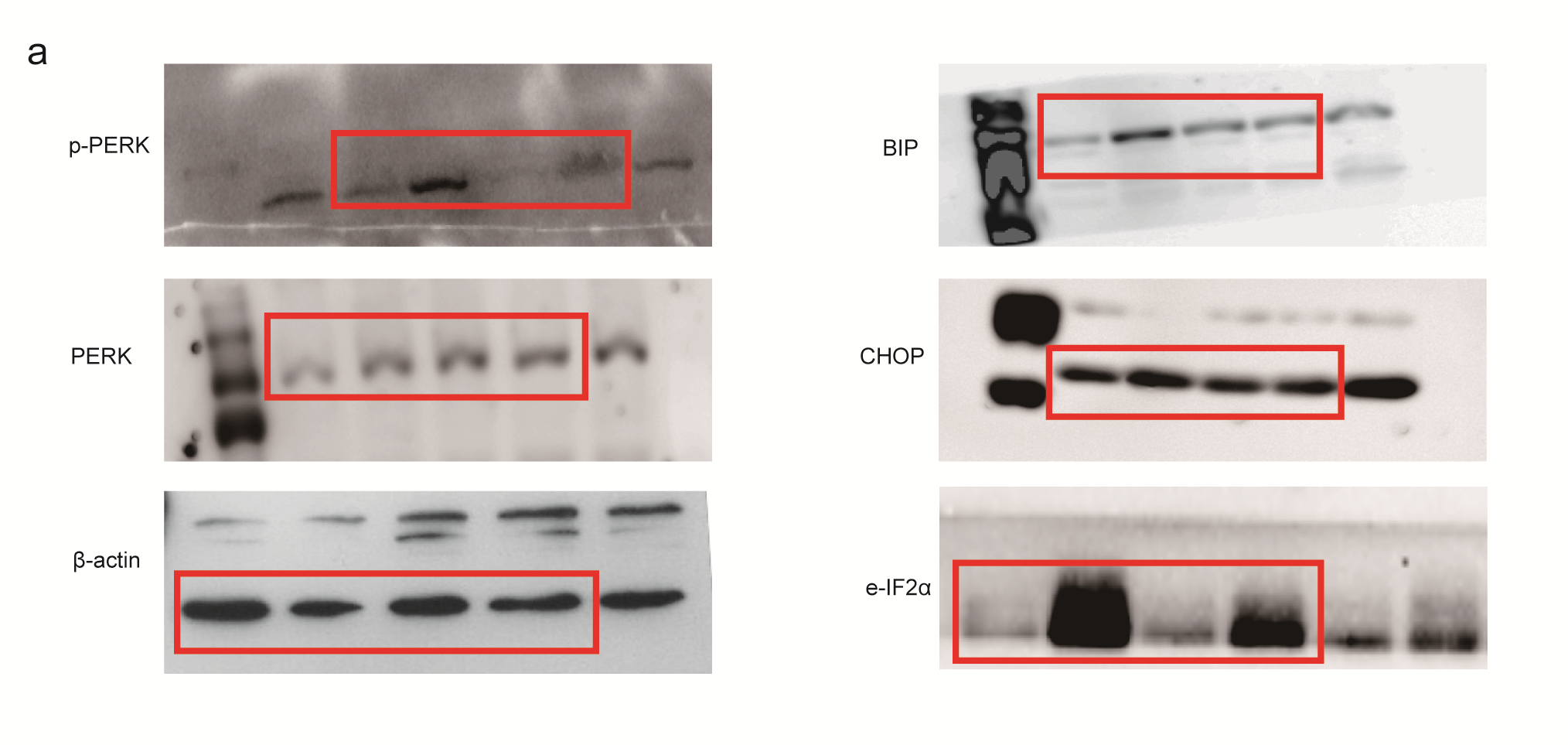


Fig S2: Full blots. Full blots of cropped blots shown in Fig 2. D


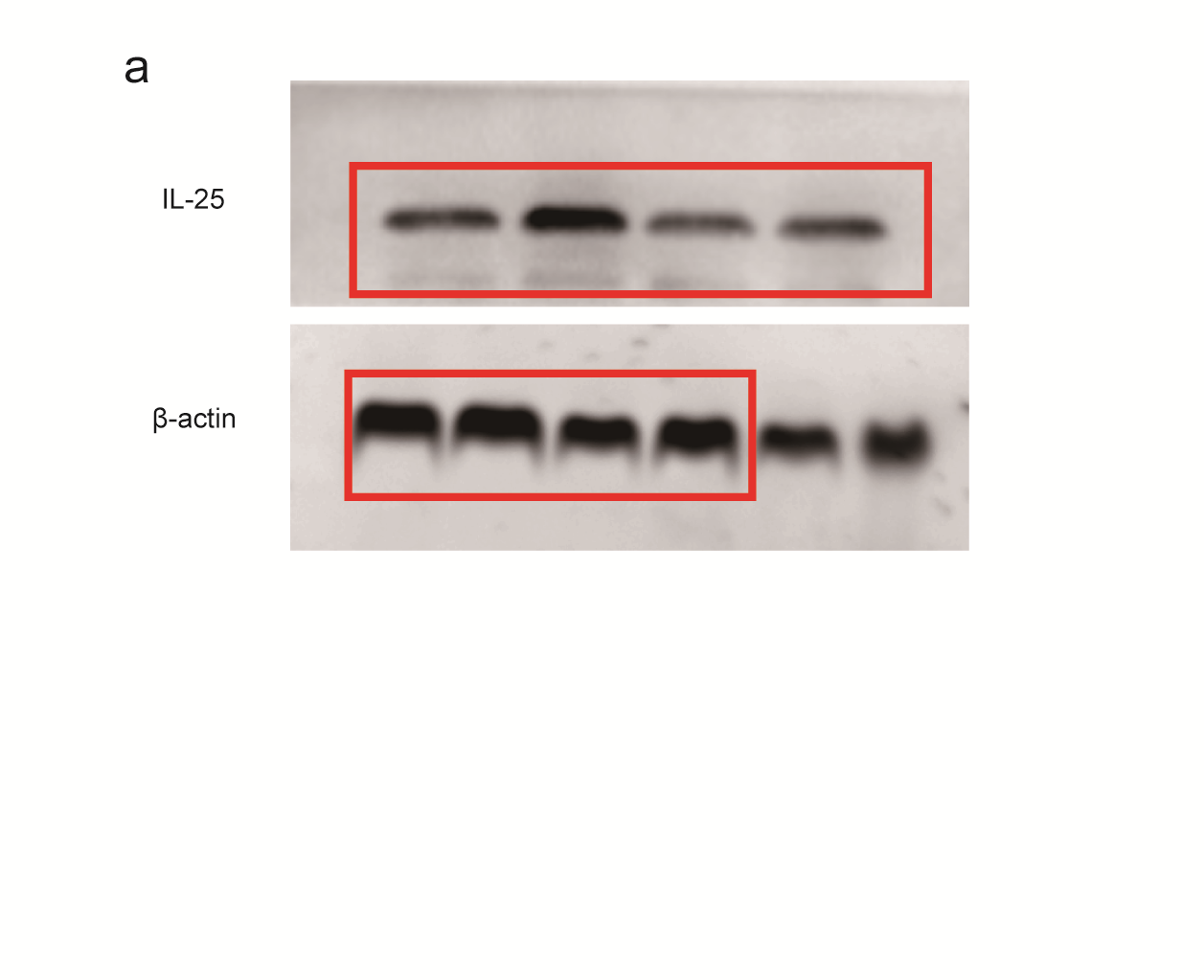


Fig S3: Full blots. (a) Full blots of cropped blots shown in Fig 3. d


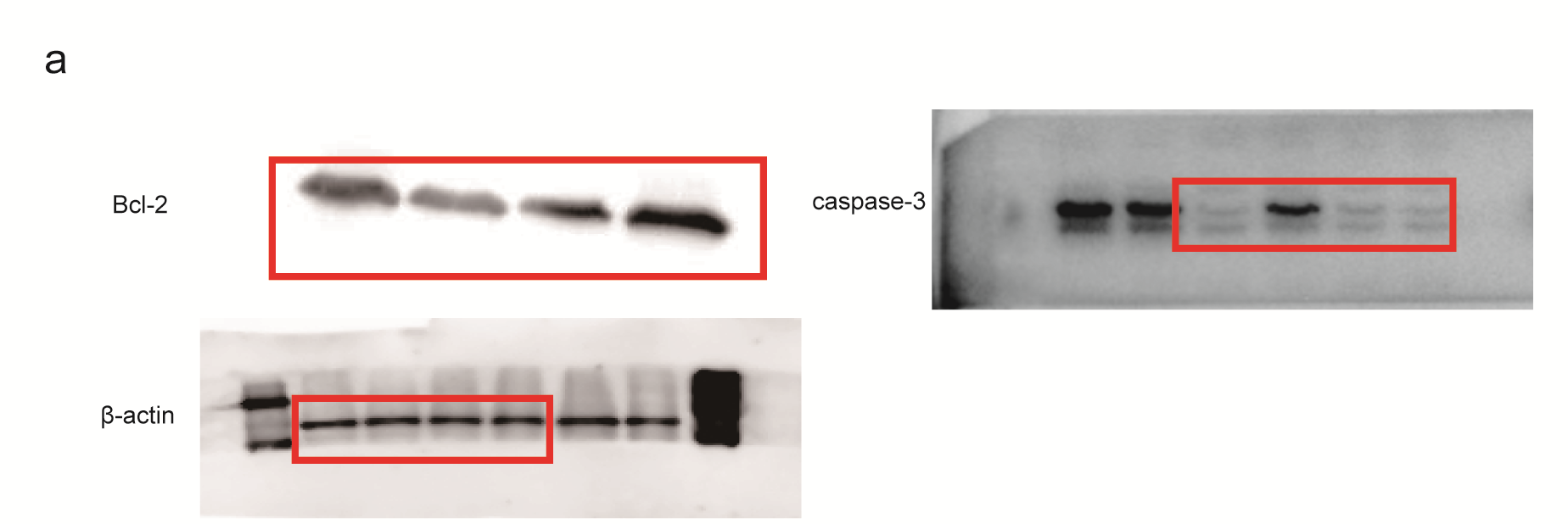


Fig S4: Full blots. (a) Full blots of cropped blots shown in Fig 4. e


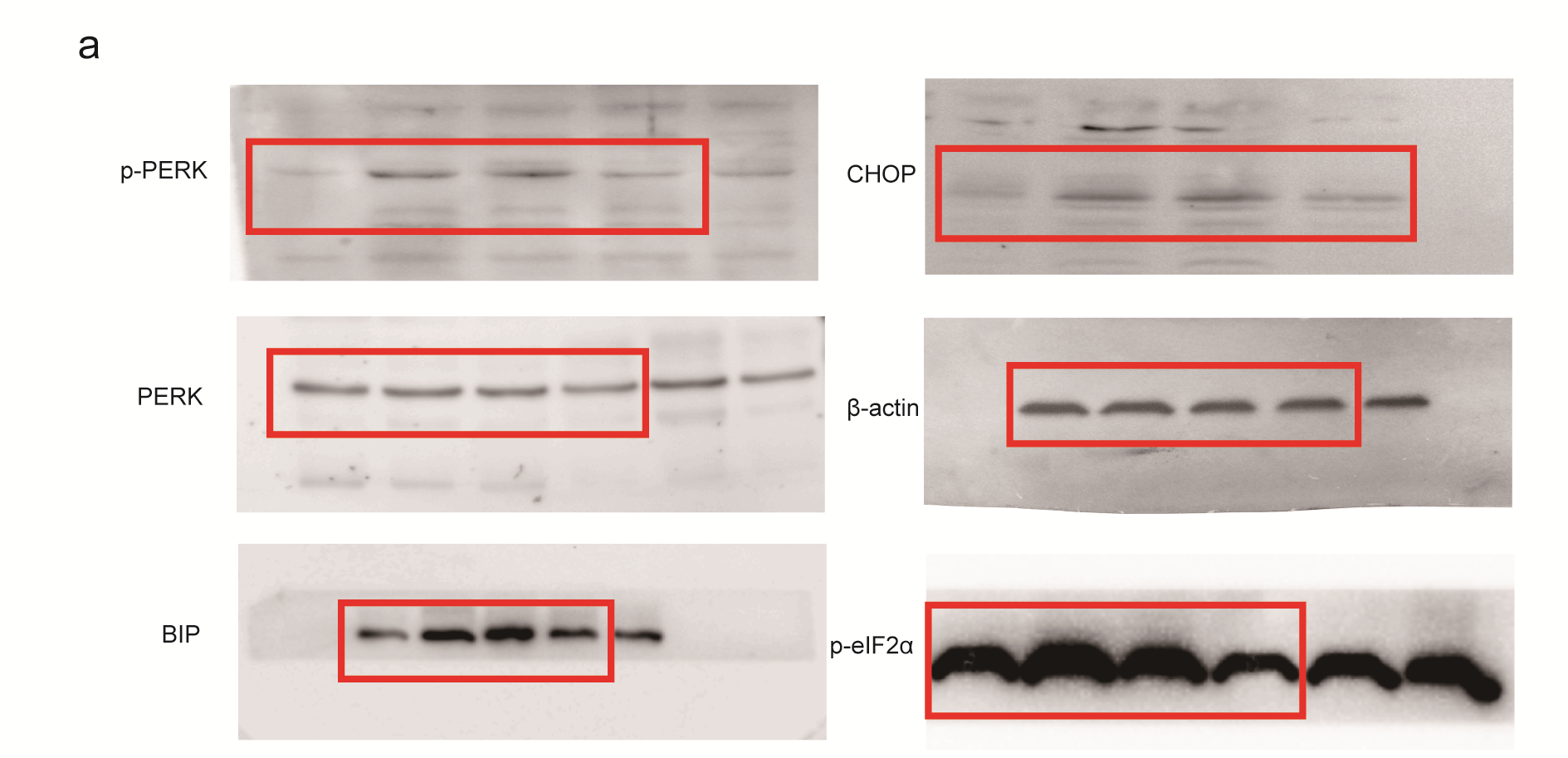


Fig S5: Full blots. (a) Full blots of cropped blots shown in Fig 6. i


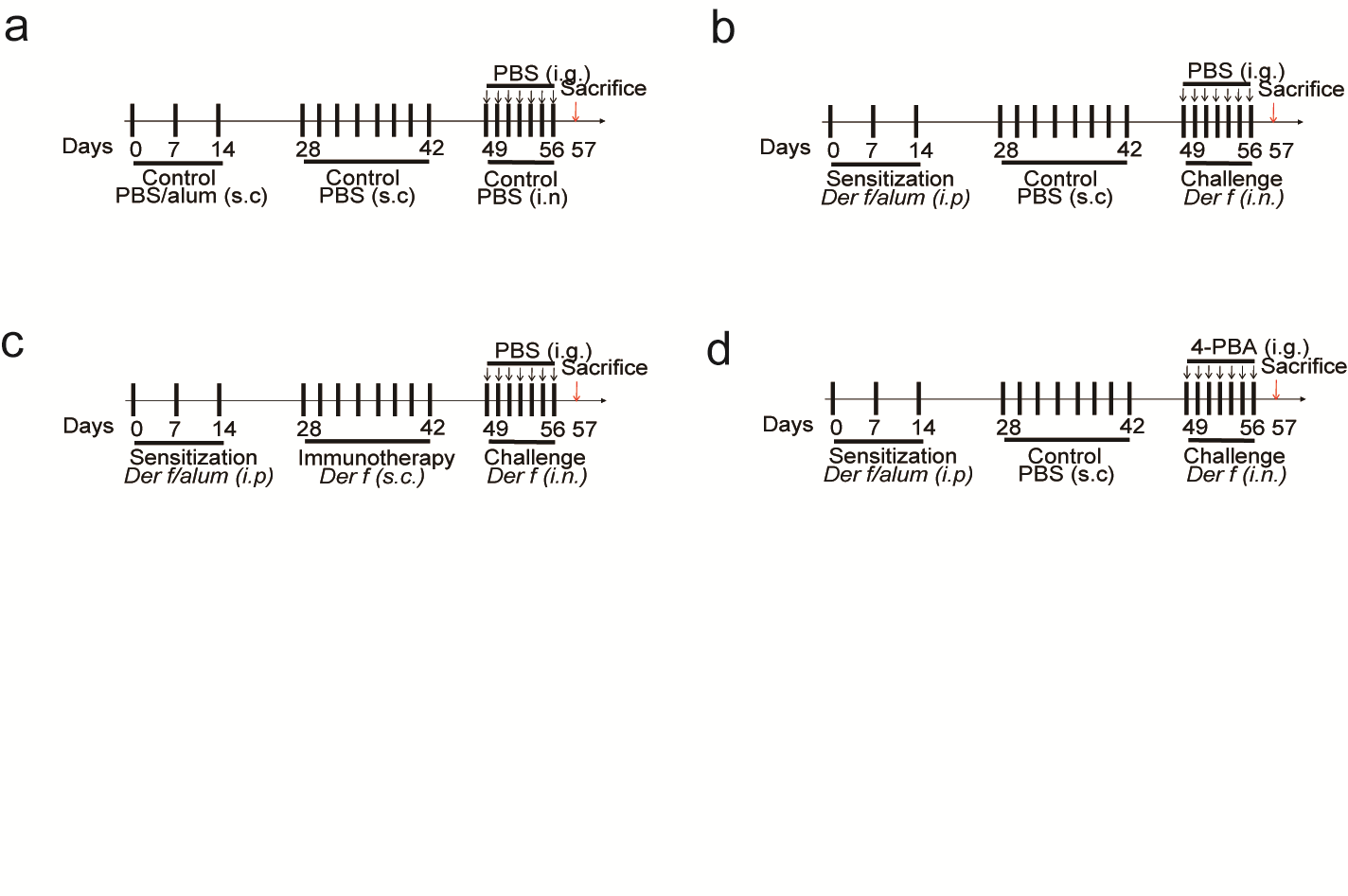


Fig S6: Experimental outlines for each group. (a-d) Experimental outlines for PBS mice, *Der f* mice, SIT mice, 4-PBA mice separately.
